# Supplementary material for: Relationship Between Effective Dose, Alternative Metrics, and SSDE: Experiences with Two CT Dose-Monitoring Systems
Source: Diagnostics (Basel). 2025 Jun 28;15(13):1654. doi: 10.3390/diagnostics15131654 (PMC12249074; doi:10.3390/diagnostics15131654)
Supplement: Supplementary file 1 [file diagnostics-15-01654-s001.zip › diagnostics-3651214-supplementary.pdf]

par	group variable	count	median	mean	std	min	max
DLP_dw	Abdomen	13082	1497	1752	1103	2	8134
	Chest	40969	512	981	1032	1	6316
	Head	24017	984	1336	1155	1	10263
	Neck	2216	1051	1157	539	10	4352
	Pelvis	197	1250	1401	569	492	3910
**Sum**		**80481**					
DLP_dms	Abdomen	13082	1497	1752	1103	2	8134
	Chest	40969	512	981	1032	1	6316
	Head	24017	984	1336	1155	1	10263
	Neck	2216	1051	1157	539	10	4352
	Pelvis	197	1250	1401	569	492	3910
**Sum**		**80481**					

Table S1. Statistical summary of DLP according to DMS and DW™ systems, and grouped by anatomical region. The unit of DLP is mGy·cm.

DLP: dose-length product, DMS: dose monitoring system; DW™: DoseWatch™

	group variable	count	median	mean	std	min	max
SSDE dw	Abdomen	19031	9.7	10.66	10.99	0	115.84
	Chest	54858	9.88	10.30	10.55	0	166.17
	Head	17458	30.82	23.29	17.82	0	107.9
	Neck	1810	26.09	26.60	21.49	0	121.56
	Pelvis	102	27.29	21.10	18.41	0.20	61.94
**Sum**		**93259**					
SSDE dms	Abdomen	19031	13.33	15.60	9.87	0.03	142.43
	Chest	54858	12.06	13.86	9.73	0.02	166.17
	Head	17458	31.88	33.74	11.76	0.04	137.31
	Neck	1810	28.68	34.40	17.01	0.07	142.88
	Pelvis	102	33.74	33.30	10.15	0.20	61.94
**Sum**		**93259**					

Table S2. Statistical summary of SSDE, grouped by anatomical region and the DMS and the DW™ systems. The unit is mGy.

par	group variable	count	median	mean	std	min	max
ED_dw	Abdomen	14541	21.01	25.13	17.18	0.92	124.46
	Chest	40810	7.54	14.23	14.93	0.19	92.03
	Head	22051	1.72	1.89	0.93	0	14.56
	Neck	1378	4.74	5.94	3.09	0	49.55
	Pelvis	603	9.09	11.16	8.08	0.38	50.33

**Sum**		79383					
ED_{DMSdms}	Abdomen	14541	20.11	24.03	16.55	0	122.74
	Chest	40810	6.69	13.87	15.34	0	103.51
	Head	22051	1.91	2.22	1.85	0	64.49
	Neck	1378	5.01	6.23	3.43	0	57.47
	Pelvis	603	10.4	12.46	8.81	0	56.46
**Sum**		79383					
SED_{DMS}	Abdomen	14541	20.36	21.82	13.46	0.0	99.93
	Chest	40810	6.36	12.80	13.59	0	86.24
	Head	22051	1.85	2.13	1.68	0	54.16
	Neck	1378	4.74	5.94	3.09	0	49.55
	Pelvis	603	9.45	11.54	7.51	0	47.03
**Sum**		79383					

Table S3. The table of statistical results of ED data is grouped by the DMS and the DW™ systems, as well as the anatomical region. The unit is mSv.

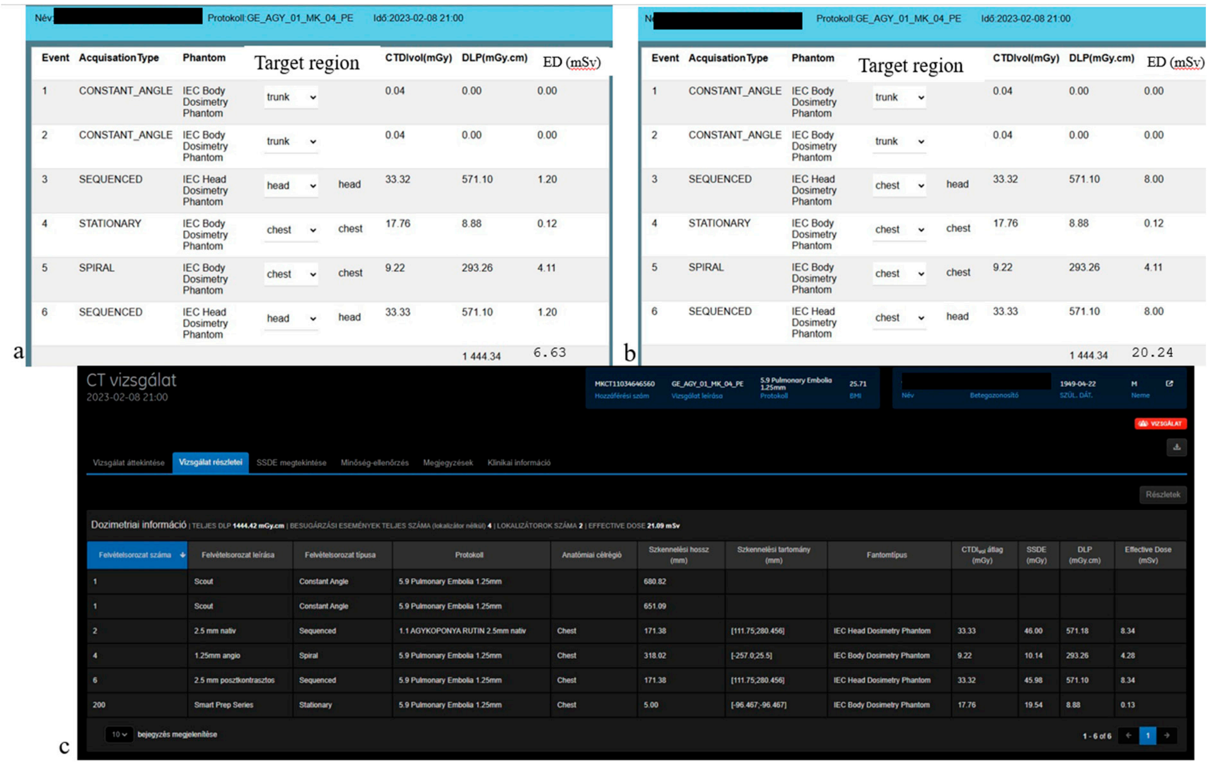

Figure S1. Representative figure of dose monitoring systems. Panels *a* and *b* present the dose report generated by the DMS of a representative study, which includes target regions and dose metrics (CTDI, DLP, ED) for each series. The DMS automatically populates the target region and remains manually editable. Panel c displays the dose report of the same study by the DW™, which provides similar data; however, the target region is fixed—non-editable—and remains the same across all series. "Panel *a*" displays the correct series-specific target regions corresponding to the actual examinations, whereas "Panel *b*" shows the target regions changed according to the DW™ system identified. The total (the study-related) effective doses are as follows: ED_{DMS}: 6.63 mSv (Panel a),

EDDMS: 20.24 mSv (Panel b), and EDDW: 21.09 mSv (Panel c). The discrete difference between the total doses (20.24 vs. 21.09) is explained by the distinct f factors used in DW™ and DMS.

CTDI: CT dose index; DLP: dose-length product; ED: effective dose; DMS: dose monitoring system; DW™: DoseWatch™

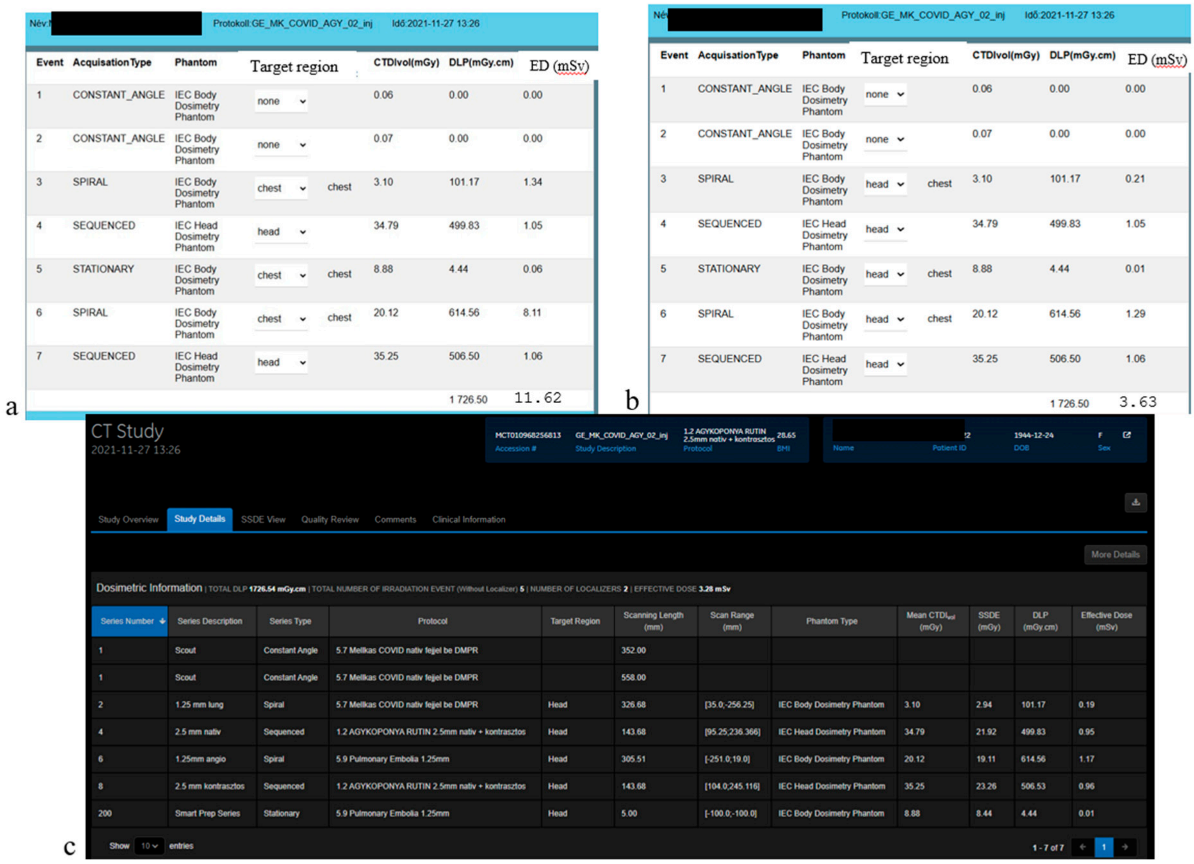

Figure S2. Representative figure of dose monitoring systems. Panels *a* and *b* present the dose report generated by the DMS of a representative study, which includes target regions and dose metrics (CTDI, DLP, ED) for each series. The DMS automatically populates the target region and remains manually editable. Panel c displays the dose report of the same study by the DW™, which provides similar data; however, the target region is fixed—non-editable—and remains consistent across all series. "Panel *a*" displays the correct series-specific target regions corresponding to the actual examinations, whereas "Panel *b*" shows the target regions changed according to the DW™ system identified. The total (the study-related) effective doses are as follows: EDDMS: 11.62 mSv (Panel a), EDDMS: 3.63 mSv (Panel b), and EDDW: 3.28 mSv (Panel c).

CTDI: CT dose index; DLP: dose-length product; ED: effective dose; DMS: dose monitoring system; DW™: DoseWatch™

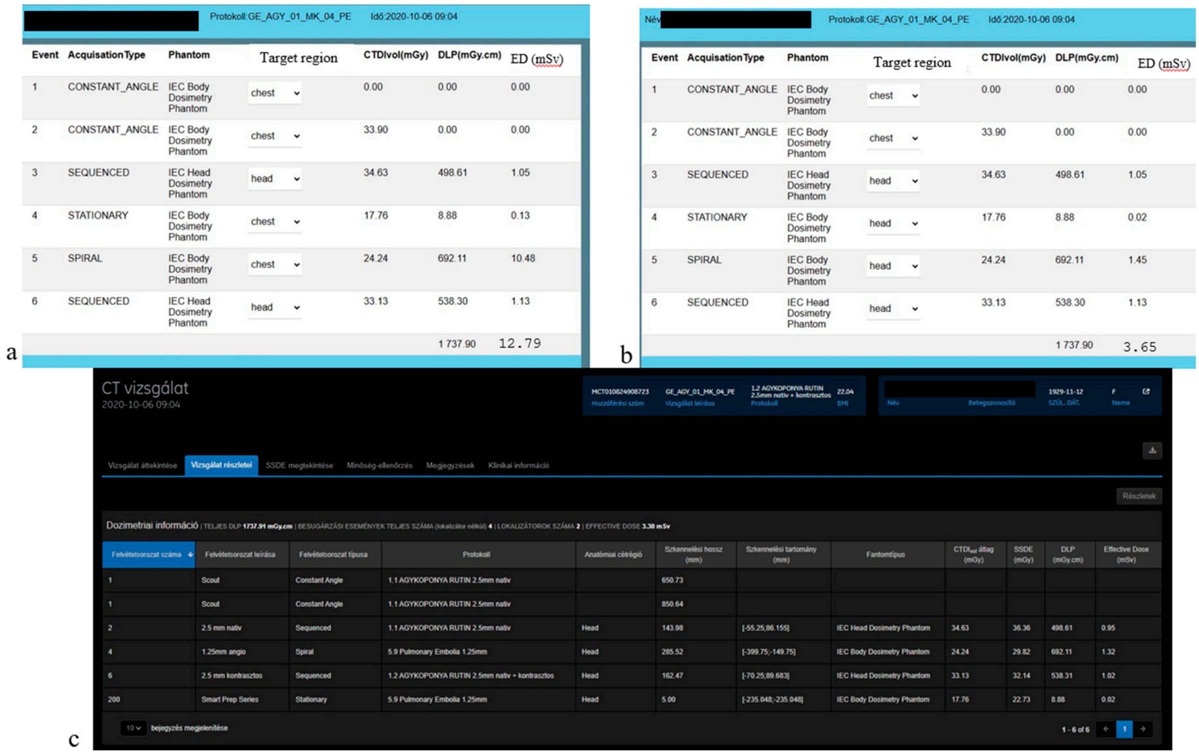

a

[redacted] Protokoll:GE_AGY_01_MK_04_PE Idő:2020-10-06 09:04

Event	AcquisationType	Phantom	Target region	CTDIvol(mGy)	DLP(mGy.cm)	ED (mSv)
1	CONSTANT_ANGLE	IEC Body Dosimetry Phantom	chest	0.00	0.00	0.00
2	CONSTANT_ANGLE	IEC Body Dosimetry Phantom	chest	33.90	0.00	0.00
3	SEQUENCED	IEC Head Dosimetry Phantom	head	34.63	498.61	1.05
4	STATIONARY	IEC Body Dosimetry Phantom	chest	17.76	8.88	0.13
5	SPIRAL	IEC Body Dosimetry Phantom	chest	24.24	692.11	10.48
6	SEQUENCED	IEC Head Dosimetry Phantom	head	33.13	538.30	1.13
					1 737.90	12.79

b

Név [redacted] Protokoll:GE_AGY_01_MK_04_PE Idő:2020-10-06 09:04

Event	AcquisationType	Phantom	Target region	CTDIvol(mGy)	DLP(mGy.cm)	ED (mSv)
1	CONSTANT_ANGLE	IEC Body Dosimetry Phantom	chest	0.00	0.00	0.00
2	CONSTANT_ANGLE	IEC Body Dosimetry Phantom	chest	33.90	0.00	0.00
3	SEQUENCED	IEC Head Dosimetry Phantom	head	34.63	498.61	1.05
4	STATIONARY	IEC Body Dosimetry Phantom	head	17.76	8.88	0.02
5	SPIRAL	IEC Body Dosimetry Phantom	head	24.24	692.11	1.45
6	SEQUENCED	IEC Head Dosimetry Phantom	head	33.13	538.30	1.13
					1 737.90	3.65

c

CT vizsgálat
2020-10-06 09:04

MCT010824908723 (Hozzáférési szám) | GE_AGY_01_MK_04_PE (Vizsgálat leírása) | 1.2 AGYKOPONYA RUTIN 2.5mm natív + kontrasztos (Protokoll) | 22.04 (BMI)

Név [redacted] Betegazonosító | 1929-11-12 (SZÜL. DÁT.) | F (Neme)

Vizsgálat áttekintése | Vizsgálat részletei | SSDE megtekintése | Minőség-ellenőrzés | Megjegyzések | Klinikai információ

Részletek

Dozimetriai információ | TELJES DLP 1737.91 mGy.cm | BESUGÁRZÁSI ESEMÉNYEK TELJES SZÁMA (lokalizátor nélkül) 4 | LOKALIZÁTOROK SZÁMA 2 | EFFECTIVE DOSE 3.30 mSv

Felvételsorozat száma	Felvételsorozat leírása	Felvételsorozat típusa	Protokoll	Anatómiai célrégió	Szkennelési hossz (mm)	Szkennelési tartomány (mm)	Fantomtípus	$CTDI_{vol}$ átlag (mGy)	SSDE (mGy)	DLP (mGy.cm)	Effective Dose (mSv)
1	Scout	Constant Angle	1.1 AGYKOPONYA RUTIN 2.5mm natív		650.73						
1	Scout	Constant Angle	1.1 AGYKOPONYA RUTIN 2.5mm natív		850.64						
2	2.5 mm natív	Sequenced	1.1 AGYKOPONYA RUTIN 2.5mm natív	Head	143.98	[-55.25;86.155]	IEC Head Dosimetry Phantom	34.63	36.36	498.61	0.95
4	1.25mm angio	Spiral	5.9 Pulmonary Embolia 1.25mm	Head	285.52	[-399.75;-149.75]	IEC Body Dosimetry Phantom	24.24	29.82	692.11	1.32
6	2.5 mm kontrasztos	Sequenced	1.2 AGYKOPONYA RUTIN 2.5mm natív + kontrasztos	Head	162.47	[-70.25;89.683]	IEC Head Dosimetry Phantom	33.13	32.14	538.31	1.02
200	Smart Prep Series	Stationary	5.9 Pulmonary Embolia 1.25mm	Head	5.00	[-235.048;-235.048]	IEC Body Dosimetry Phantom	17.76	22.73	8.88	0.02

10 bejegyzés megjelenítése 1 - 6 of 6

Figure S3. Representative figure of dose monitoring systems. Panels *a* and *b* present the dose report generated by the DMS of a representative study, which includes target regions and dose metrics (CTDI, DLP, ED) for each series. The DMS automatically populates the target region and remains manually editable. Panel c displays the dose report of the same study by the DW^{TM}, which provides similar data; however, the target region is fixed—non-editable—and remains consistent across all series. "Panel *a*" displays the correct series-specific target regions corresponding to the actual examinations, whereas "Panel *b*" shows the target regions changed according to the DW^{TM} system identified. The total (the study-related) effective doses are as follows: ED_{DMS}: 12.79 mSv (Panel a), ED_{DMS}: 3.65 mSv (Panel b), and ED_{DW}: 3.30 mSv (Panel c).

CTDI: CT dose index; DLP: dose-length product; ED: effective dose; DMS: dose monitoring system; DW^{TM}: DoseWatch^{TM}
